# Bringing Gene Therapies for HIV Disease to Resource-Limited Parts of the World

**DOI:** 10.1089/hum.2020.252

**Published:** 2021-01-18

**Authors:** Joseph M. McCune, Emily H. Turner, Adam Jiang, Brian P. Doehle

**Affiliations:** HIV Frontiers, Global Health Innovative Technology Solutions, Bill & Melinda Gates Foundation, Seattle, Washington, USA.

## Introduction

Despite the fact that 59% of people living with HIV (PLHIV) currently achieve viral suppression on antiretroviral therapy (ART), recent gains in controlling the global HIV/AIDS epidemic may be threatened: key HIV incidence rates are declining only modestly, the sustainability of programs to expand ART remains unclear, and the “youth bulge” in sub-Saharan Africa contributes to a growing at-risk population.^1^ Although much effort has been devoted to prevention interventions, these face major technical and/or implementation challenges.

A complementary approach is a safe, effective, and durable intervention that completely eliminates HIV infection (“eradication”) or that suppresses viremia in the absence of ART (“remission”) (both of these states are referred to as an “HIV cure” herein). Although a daunting goal, the scientific basis is clear: long-term remission if not eradication has been observed in the “Berlin patient”^2^ as well as the “London patient”^3^ following transplantation of bone marrow progenitor cells lacking the viral coreceptor, CCR5; and durable remission occurs in tens of thousands of PLHIV (so-called “Elite Controllers”), some of whom (“Exceptional Elite Controllers”) may have eliminated their infections through natural immunity.^4,5^

Ongoing work over the past decade suggests that HIV cure might be induced by some interventions, alone or in combination, including provision of broadly neutralizing antibodies, generation of effective antiviral CD8^+^ T cell responses, and knockout of the viral coreceptor, CCR5.^6^ Little is known, however, about the nature and vulnerabilities of the rebound-competent viral reservoir that persists despite ART and about the immunologic control of virus in the absence of ART; today's “best bets,” in other words, must still be viewed as long shots.

**Figure 1.**
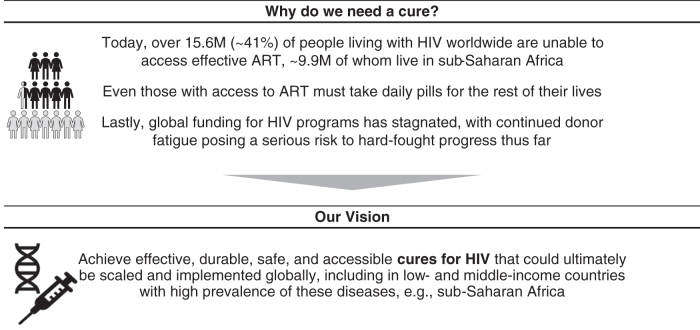
The vision for the HIV Frontiers Program.

Initiated by the Bill & Melinda Gates Foundation in 2019, the HIV Frontiers Program aims to move work on HIV cure toward interventions that will ultimately be available to all, most especially those in resource-limited parts of the world where the prevalence of disease is high (Fig. 1). It starts with the premise that the journey will be long (15–25 years) and that it will ultimately yield a “single-shot cure,” that is, a product that is delivered percutaneously (*in vivo*) in a single encounter, safely, and effectively modifying selected cells in the body so that viral replication and spread are suppressed and reinfection blocked.

This aspirational goal is likely to be realized through a series of progressive interventions that move from combination therapies provided over a longer duration of time to those in which cells are modified outside of the body (*ex vivo*) before reinfusion.^7^ The work will build on current knowledge to advance through a series of technical and practical hurdles while also gathering new knowledge to best design a curative intervention for HIV and to determine whether and how it might be used.

To get to the point of having a “single-shot cure” for HIV in hand, two interlocking areas of focus are being pursued:

### Current best bets

To minimize the expected time to impact, investments are being made in all of the necessary elements of a “single-shot” HIV cure in parallel. As a critical enabler, the Program is leveraging the considerable interest and resources in biotech/pharma companies that are now developing *ex vivo* genetic and cell-based interventions; uniquely among ongoing efforts, it intends to shift the emphasis of such interventions to delivery *in vivo*, an approach that is much more likely to benefit those in resource-limited parts of the world.

Early forms of the “single-shot” cure would tap current “best bets” (*i.e.*, administration of two or more broadly neutralizing antibodies [bNAbs], induction of a durable T cell response against HIV, and CCR5 knockout), quickly pivoting to others should they arise, and asking the questions: can these interventions be delivered efficiently and safely to appropriate cell populations *in vivo*; if so, can they be associated with methods to detect their failure; and, importantly, is there a viable pathway for product development and distribution in sub-Saharan Africa? Incremental steps are being taken to maximize the likelihood of success, with development and validation of novel approaches for targeting and editing selected populations of cells (*e.g.*, hematopoietic stem cells, CD4^+^ T stem central memory cells, and B cells) *in vivo*.

At the outset, this work is taking advantage of genetic “cures” for sickle cell disease that are now in hand, with early results indicating that substantial clinical benefit can be obtained with even incomplete correction of the hemoglobin S genotype (either by editing the hemoglobin S allele or by upregulating hemoglobin F) in hematopoietic stem cells *ex vivo*.^8^ Successful efforts to modify hematopoietic stem cells *in vivo* to result in similar corrections might form a pathway to the *in vivo* introduction of modifications aimed at HIV cure. If successful, the ultimate product will be an inexpensive composition that is easily and safely delivered, and designed to effect a durable HIV cure for all; en route, interventions providing benefit for those with sickle cell disease (as well as other hemoglobinopathies) should predictably arise.

### The HIV reservoirs consortium

In this program, a consortium of academic laboratories has been established to define the biology of the rebound-competent reservoir of HIV *in vivo* and, in particular, to discover circulating nonviral biomarkers that can be used to monitor it over time. A strategically focused, multidisciplinary team effort is carrying out studies in people living with HIV (PLHIV) in resource-limited parts of the world, as well as in nonhuman primate models that recapitulate relevant aspects of human HIV infection and in which the reservoir can be systematically perturbed with interventions that could not be used in humans. Using state-of-the-art assays, it is hoped that circulating nonviral biomarkers for the rebound-competent reservoir will be discovered in the nonhuman primate, crossvalidated in the human, and assessed for their ability to define the size and quality of the rebound-competent reservoir while on antiretroviral therapy (ART), and the time to viral rebound once ART is discontinued.

Of note, the HIV Frontiers Program presupposes the will to assume and to share significant risk. Substantial new financial resources and a sustained commitment will be required to pursue the multiple components of a “single-shot” HIV cure in parallel and to simultaneously launch the HIV Reservoirs Consortium. Such resources and commitment will not arise from a single source; rather, partnerships must be formed and strategic priorities set. This review will outline some of the steps that are being taken to reach these goals.

## The Need for an HIV Cure

Even though dramatic advances have been made in the diagnosis, treatment, and prevention of HIV infection, the pandemic continues unabated. Especially in countries that are resource limited, alarming rates of new infections persist, and it is unlikely that sufficient funds will become available for universal testing, treatment, and follow-up care. Even should funding emerge, life-long adherence to suppressive ART is difficult for many. In the worst-case scenario, funding for treatment and care will remain the same or even diminish, and with an anticipated “youth bulge” and increasing resistance to ART,^1^ an uncontrolled epidemic may possibly even grow.^6,9^

To avoid this, we must stop acquisition of HIV by those at risk. One way to do this is by primary prevention, for example, with a vaccine, long-lasting pre-exposure prophylaxis, voluntary medical male circumcision, barrier methods, and structural and behavioral changes. Another way is to prevent transmission from PLHIV, for example, by an intervention that lowers the circulating viral load to a point at which transmission is unlikely (*e.g.*, <50 copies/mL).^7,10^ Such an intervention would have particular impact when targeted to select populations that are now untreated and likely to transmit virus. The HIV Frontiers Program is focused on the discovery and development of an intervention of this type that could be used anywhere in the world. Notably, this is an effort aimed to be complementary with ongoing work focused more squarely on treatment of disease or prevention of infection.

## Definition of an HIV Cure

To achieve impact on the epidemic, an HIV cure does not need to completely eradicate HIV in an individual (a state that can only be inferred) but, rather, needs to durably maintain viremia at levels that prevent disease progression, allow for good health, and nearly eliminate the possibility of transmission. To be universally accessible, an HIV cure must be deployable in areas of the world where health care infrastructure is limited. It must also place acceptable intervention and monitoring requirements on the patient, and must be effective against ART-resistant viruses.^6^

Putting this together, an idealized HIV cure appropriate for all parts of the world might be viewed as a safe intervention with no widespread contraindications that is delivered percutaneously in a single outpatient encounter (referred to, in this study, as a “single-shot”) and that durably (*e.g.*,>3 years) reduces viral load to <50 copies/mL.^7,11^ Notably, it must maintain viral suppression even if the individual is exposed to reinfection.^9^ Assuming upfront that it will fail over time in at least some individuals, it must also incorporate a means by which rebound viremia can be easily detected and, ideally, readministration of the cure intervention will re-establish remission. The acceptable cost of the intervention will in large part be dependent on the durability of its effect; by some predictions, the amortized cost of the single-shot cure, plus the ongoing rebound monitoring should be less than $100 per year.^12^ These and other considerations related to an evolving progression of target product profiles for an HIV cure are more completely discussed in Lewin *et al.*^7^

## Hurdles Confronting an HIV Cure

Although the “cure” agenda has been vigorously advanced by the academic research community and to a more limited degree by biotech/pharma, scientific progress has been stymied by our relative lack of understanding of the biology of the rebound-competent viral reservoir, which persists in the face of suppressive ART. It is difficult to quantify with accuracy, we do not know how it is sustained, and we have only emerging clues about how it is sometimes naturally contained by the immune system.^13^ Although it clearly changes as a function of time after infection, studies in humans have been mostly limited to a narrow demographic of infected individuals,^14^ providing little insight into elements of heterogeneity related to age, gender, race, or concomitant conditions (*e.g.*, other chronic coinfections).

Technically, it has been hard to clarify these unknowns because the viral reservoir resides in tissues (*e.g.*, lymphoid organs, the central nervous system, and the gut) that cannot be easily or comprehensively accessed in PLHIV. Notwithstanding decades of observations in mice and humans suggesting that cells which circulate in the peripheral blood represent but a small fraction of those residing within tissues, with cell/cell interactions and qualitative functional features that are totally distinct, most of our knowledge of the virology and immunology of HIV disease derives from studies on CD4^+^ T cells in the peripheral blood. We are, in short, ignorant of some of the most basic features of the rebound-competent reservoir and the immune response against it.

## Successes, Failures, and Current Efforts in the HIV Cure Arena

Nevertheless, work on HIV cure continues and progress is being made. Repeated observations of a small number of people indicate that viral control is possible. Widely heralded has been the true “N of 1” of the HIV cure world: the “Berlin patient” who received several bone marrow transplants with hematopoietic stem cells deficient for the viral coreceptor, CCR5.^3^ Notably, this intervention was prompted by earlier studies showing that natural mutations in CCR5 (*e.g.*, the CCR5Δ32 genotype that is relatively common in Northern Europe) confer resistance to HIV infection.^15,16^ Of note, there is now a second individual (the “London patient”) who was provided a less-aggressive course of conditioning and who appears to have been cured of HIV, suggesting that this approach may become more safely accessible to others in the future.^3^

It is also the case that some PLHIV are able to naturally control HIV after infection, either spontaneously (“Elite Controllers” and “Exceptional Elite Controllers,” the latter defined as PLHIV with no plasma viremia after at least 10 years of documented infection in the absence of ART, apparently eliminating it through natural immunity)^4,5^ or after a period of suppressive ART (“Posttreatment Controllers”),^17^ apparently because they are able to mount effective antiviral CD8^+^ T cell and/or NK cell responses against HIV. In like fashion, the provision of several bNAbs to SHIV-infected monkeys^18^ and to PLHIV on suppressive ART^19,20^ induced antiviral CD8^+^ T cell responses and prolonged viral remission off ART in some. Also recently, protection of CD4^+^ T stem central memory cells from HIV infection by CCR5 gene editing has been associated with a higher frequency of antiviral CD8^+^ T cells, increased CD4^+^ T cells counts, and decreased viral reservoir measurements in chronically HIV-infected individuals (R. Sekaly, pers. comm.). Importantly, these latter two findings are both consistent with the hypothesis that suppressive antiviral T cell responses can be newly elicited in PLHIV and might be especially potent mediators of durable ART-free remission.

For every such hint of a possible route toward a cure, there have been multiple incorrectly interpreted observations (some recent examples: the use of anti-α4β7 antibodies to cure SIV infection *in vivo*^21,22^ and the definition of CD32 as a specific marker for the rebound-competent reservoir^23–27^) or instructive failures (*e.g.*, the inability of early ART initiation to cure in the “Mississippi baby”^28^ and the “San Francisco patient,”^29^ the rebound of virus from the “Boston patients” who were myeloablated as part of care for their malignancies,^30^ and X4 HIV escape in the “Essen patient” after a CCR5Δ32 bone marrow transplant^31^). It has also become increasingly evident that the rebound-competent reservoir is vast^32^ and that tissue-based immune responses against it might be crippled, especially in those who are treated at later time points after infection.

Work is nonetheless ongoing to test a number of strategies for an HIV cure. As detailed in several recent reviews, broad categories now being pursued include:

### HIV-specific immune enhancement

Hoping to replicate the ability of “Elite/Exceptional Elite Controllers” and “Posttreatment Controllers” to suppress HIV viremia in the absence of ART, much work is focused on the development of therapeutic T cell vaccines to enhance HIV-specific T cell responses in those who are infected or to redirect the T cell response to drive mutations that impair viral fitness.^4,33,34^ Also being explored is the use of CAR T cells to enable strong, antigen-specific, MHC-unrestricted T cell responses.^35,36^ More recently, administration of several bNAbs has been found to not only block viral spread but to also lead to durable (>1 year) viral remission in some,^19^ possibly because an indirect CD8^+^ T cell “vaccinal effect” is induced when bNAb/HIV immune complexes are internalized by antigen-presenting cells.^37,38^

### Immune modulation

To further enhance durable T cell immunity, a number of approaches are being pursued to reverse the immune dysfunction induced by progressive HIV disease. Thus, immune checkpoint blockers such as pembrolizumab (anti-PD1) and ipilimumab (anti-CTLA4) are being evaluated for their ability to augment HIV-specific functional immune responses,^39–41^ vedolizumab (anti-α4β7) for its ability to reduce trafficking of susceptible CD4^+^ T cells to the gut HIV reservoir,^42^ and the IL-15 superagonist ALT-803/N-803 for its ability to improve trafficking of CD8^+^ T cells to lymphoid tissue B cell follicles.^43,44^ A variety of other agents (*e.g.*, sirolimus, an IL-21 superagonist, and anti-IFNα/β receptor antibodies) are also being tested for their ability to reduce HIV-associated inflammation, hoping to thereby improve HIV-specific immune responses.^45–47^

### Targeting the HIV replication cycle

These approaches are designed to either inhibit infection of CD4^+^ target cells (*e.g.*, by transplantation of bone marrow stem cells from a CCR5Δ32 donor, knockout of the endogenous CCR5 locus, and/or expression of antagonists that prevent viral entry into potential target cells)^6,48,49^ or to reverse latency and increase transcription of HIV (*e.g.*, by provision of latency reversal agents such as histone deacetylase inhibitors, Toll-like receptor agonists, or disulfiram),^50^ thereby effecting virus- or immune-mediated cytolysis of infected cells (“kick-and-kill”). Conversely, it might be possible to “block-and-lock” the viral genome, for example, by inhibition of Tat with didehydro-cortistatin A or with the use of interfering RNAs to silence the viral promoter or cause degradation of complementary viral mRNA.^6,45^ Finally, gene editing strategies (*e.g.*, with CRISPR/Cas9 and the Brec1 recombinase) are being employed to activate or inactivate the integrated proviral genome *in situ.*^51,52^

Data about the impact of some, if not all, of these approaches should be forthcoming in the next 2–4 years from a diverse array of interventional and observational studies that are now ongoing, although at an early stage and with a narrow demographic of participants and small sample sizes.^14^

## Limitations of Current Funding Mechanisms

Although HIV cure research has attracted substantial resources and much energy since it was formally launched over a decade ago,^53^ there has been little consideration given to interventions that might be used in resource-limited parts of the world and no coordinated plan to discover circulating nonviral biomarkers to qualitatively and quantitatively define the rebound-competent reservoir in those on or discontinuing ART. Given the absence of such biomarkers and the presence of other daunting technical challenges as well as concerns about short-term return on investment, biotech/pharma has more or less shied away from this work and the few companies with serious programs will not be easily able to prioritize products for resource-limited countries.

Meanwhile, even though the NIH and organizations such as amfAR have devoted considerable resources to academic laboratories to discover and develop cures for HIV and to more deeply study the biology of the rebound-competent reservoir, the incentive structure of academia is not well suited for the sustained support of multidisciplinary team efforts of high risk, particularly those not also associated with a downstream profit motive that might attract the attention of biotech/pharma. In aggregate, the motivating factors and resources required to bring curative interventions for HIV to resource-limited parts of the world would appear to require new models for discovery, innovation, and risk-sharing partnership.

## Strategy of the HIV Frontiers Program

The HIV Frontiers Program has been devised as a first step toward building these models. It aims to take a comprehensive approach toward developing an effective, durable, safe, and affordable single-shot HIV cure that could be used anywhere in the world, envisioning two interlocking areas of focus: one that would explore the Current Best Bets for HIV cure and another that would create an HIV Reservoirs Consortium to understand the biology of the rebound-competent reservoir and circulating nonviral biomarkers of it.

## Current Best Bets

**Figure 2.**
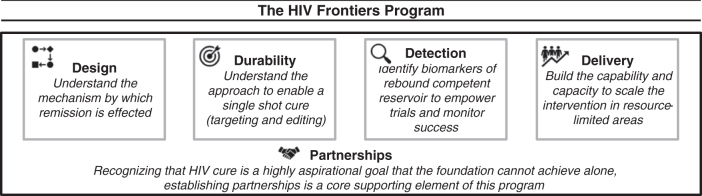
The key elements of the HIV Frontiers Program.

Achieving a single-shot HIV cure requires integration of four key elements (Fig. 2): (1) design of the cure, (2) *in vivo* delivery and durability of the design, (3) detection of the loss of remission, and (4) distribution of the intervention in sub-Saharan Africa. Rather than to await the definition of the best design for a curative intervention and to then pursue the remaining elements, the HIV Frontiers Program is advancing all in parallel. This approach is motivated by the goal of minimizing the expected timeline to impact and enabled by existing commercial interest in cell targeting and editing, diagnostics, and curing sickle cell disease.^54^ Indeed, we view the extension of promising *ex vivo* gene therapies for sickle cell disease to people in need in sub-Saharan Africa as an imperative; this exercise may also serve as a pathfinder for analogous *in vivo* gene therapy approaches for an HIV cure.

Although research on *in vivo* cellular and genetic modifications is at a very early stage, the following projections can now be reasonably made:

### Design

If the design of a broadly applicable HIV cure remains elusive, it is nonetheless possible to imagine (as described in more detail in Lewin *et al.*^7^) that there will be an evolutionary progression of curative target product profiles, moving over time from combination therapies to *ex vivo* gene therapies to *in vivo* gene therapies. Modeling of the epidemic in sub-Saharan Africa, where transmission rates remain high, suggests that an optimal curative intervention should not only suppress (or eliminate) the rebound-competent reservoir within affected individuals but also prevent infection upon re-exposure to virus.^9^

Among the approaches described above, those that now look most promising for use in resource-limited parts of the world include interventions that provide a durable effect without repeated administration, for example, extended provision of multiple bNAbs or of eCD4, and/or the induction of a long-lasting and effective antiviral immune response. Given the examples of the “Berlin patient” and the “London patient” as well as intriguing preliminary data with directed CCR5 knockout in peripheral blood cells, interventions ablating this locus might also be pursued.

These and many other approaches are being advanced, alone or in combination, into human clinical trials, data from which should be forthcoming in the next several years to more completely inform the best design for an HIV cure. Notably, however, most of these trials are being conducted in a narrow demographic (mostly older white men living in the United States, Europe, and Australia).^14^ It will accordingly be crucial to test the hypothesis that curative interventions in this demographic will be of benefit to others, especially those in resource-limited parts of the world.

### Delivery and durability

**Figure 3.**
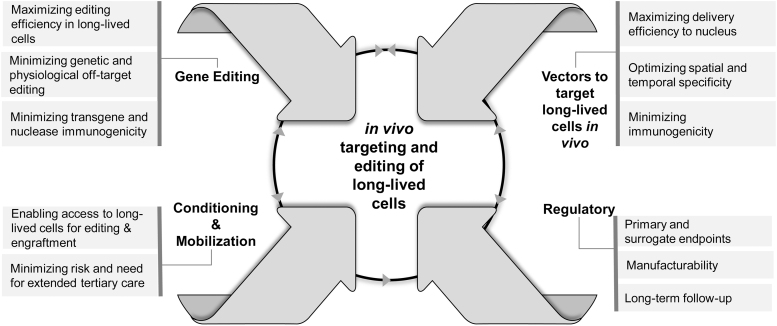
Critical considerations in the discovery, development, and clinical application of therapies targeting and editing long-lived cells *in vivo.*

Although many of the above “best bets” are being provided by *ex vivo* manipulation of cells (*e.g.*, in the case of CCR5 knockout) or repeated administration of GMP-grade protein (*e.g.*, in the case of bNAbs), rapid and sustained advances in the fields of cellular and genetic therapy, now being pursued by many companies, suggest that most could instead be introduced by *in vivo* targeting and editing of long-lived cells (Fig. 3). By example, bNAbs might be delivered to long-lived B cells as self-replicating mRNA or by stable modification of the immunoglobulin locus itself, and CCR5 might be knocked out in bone marrow-resident hematopoietic stem cells or circulating T stem central memory cells. Importantly, durable immune responses might also be generated upon induction of a “vaccinal effect” by immune complexes formed between bNAbs and HIV, or by provision of appropriate vaccines to antigen-presenting cells.

To introduce such modifications *in vivo*, it will be necessary to design vectors that can carry the appropriate editing systems to selected target cells after percutaneous (*e.g.*, intravenous or intramuscular) administration. Active work in this area^55–57^ has already highlighted the capabilities of viral (*e.g.*, AAV and lentiviral) vectors as well as ligand-directed lipid nanoparticles, ribonucleoprotein complexes, synthetic nucleocapsids, and exosomes; given commercial opportunities in areas like oncology, it is inevitable that even more options will surface in the future.

As a first step, the HIV Frontiers Program is investing in studies to ask the question: can multilineage hematopoietic stem cells, mobilized or not, be targeted by viral or nonviral vectors *in vivo* (*e.g.*, in humanized mouse models and nonhuman primates) and then edited to assume new functional traits (*e.g.*, upregulation of hemoglobin F in the case of sickle cell disease)? We envision that these proof-of-concept experiments would likely have carryover impact for modification of such cells for purposes of HIV cure (*e.g.*, introduction of genes encoding bNAbs), while also providing valuable information about the targeting and editing of other long-lived cell populations of interest, for example, T stem central memory cells and B cells.

### Detection

Anticipating that methods conferring lifelong remission will take years if not decades to perfect, it will be necessary in the interim to develop methods by which viral rebound can be reliably detected. Efforts are already being made to develop inexpensive home test kits that can be used by the individual, some of which are able to detect low viral loads (839 copies/mL) with a single finger prick.^58^ These efforts could be extended in the future to develop approaches to viral detection that are even more simple, affordable, and accurate. We also envision that they will be vastly accelerated if it is possible to define circulating nonviral biomarkers of the rebound-competent reservoir (see The HIV Reservoirs Consortium section).

### Distribution

It is unlikely that any approach involving cell targeting and editing *in vivo* will move into sub-Saharan Africa until and unless there are compelling reasons to believe that it will safely provide benefit to those in need. The most immediate motivation will be promising data for the cure of sickle cell disease. As mentioned above, several *ex vivo* approaches toward a genetic cure of sickle cell disease are now in hand, each of which is being pursued by for-profit US-based companies and the NIH. Early results indicate that substantial clinical benefit can be obtained with even incomplete correction of the hemoglobin S genotype (either upon editing the hemoglobin S allele or by upregulation of hemoglobin F).

Working in collaboration with these efforts, the technical envelope for *in vivo* delivery of analogous interventions will be explored, first in the United States and then in sub-Saharan Africa. Successful validation of targeting and editing approaches in sub-Saharan Africa (which would be its own reward) would provide early confidence that similar technical, clinical, regulatory, and introduction approaches could be taken toward HIV cure, where links between proposed target modifications and clinical success are now less certain.

We have also considered it important to initiate—sooner than later—a vigorous discussion about the ultimate implementation of curative interventions for HIV and sickle cell disease into sub-Saharan Africa. To this end, plans have been made to launch the HIV Cure Africa Acceleration Partnership, a multidisciplinary public–private partnership designed to catalyze and promote HIV cure research through engagement of diverse stakeholders and community members, convening them at an early stage to accelerate the design, socialization, implementation, and rapid adoption of HIV cure products.^59^ We imagine that it—or a similar public–private partnership—might also serve to advance curative interventions for sickle cell disease.

## The HIV Reservoirs Consortium

To be maximally effective and practical, interventions to cure HIV must be designed to suppress the rebound-competent reservoir of HIV over a relatively long period of time, for example, by engaging an immune response that destroys and/or prevents viral spread from cells harboring rebound-competent viral genomes. As in the case of staging in the treatment of cancer, it is possible that—depending on the absolute size of the reservoir and its disposition among cell types and organs of the body—different interventions will be required and/or found to be efficacious at varying stages of HIV disease progression. It is also possible that quantitative and/or qualitative aspects of the rebound-competent reservoir will be directly or indirectly affected by a number of variables, including the duration and magnitude of infection before the initiation of suppressive ART, age, gender, alterations in the mucosal microbiome, and the presence of concomitant coinfections, for example, with tuberculosis, helminthic worms, and other chronically sustained viruses.

Comprehensive definition of the rebound-competent reservoir in ART-suppressed individuals in geographically and demographically dispersed cohorts would enable assessment of possible quantitative and qualitative variations in its size and disposition, evaluation of the impact of interventions designed to suppress it, and discovery of circulating nonviral biomarker(s) that could be used to monitor it over time.

To accomplish these goals, key gaps in knowledge must be filled: (1) how can we measure the rebound-competent reservoir, (2) what are the mechanisms that sustain it *in vivo*, (3) which immune responses serve to keep it at bay, (4) what is the immune basis for natural control of HIV, (5) can this natural control be induced in the setting of ART in progressive infection, and (6) what is the best way to test, prioritize, and optimize single-shot HIV cure concepts? Some of these questions (*e.g.*, 1, 3, 5, and 6) can and should be addressed in both humans and SHIV/SIV-infected NHP, while others are only possible to definitively address in the nonhuman primate (2) or the human (4).

To move this work forward, the HIV Frontiers Program has established an “HIV Reservoirs Consortium,” coordinating the work of five teams and an aggregate of 40 laboratories for an initial period of 4 years. The primary goals of this Consortium are discovery and validation of circulating nonviral biomarkers that have predictive, monitoring, diagnostic, and/or prognostic value for HIV cure.^60^ Ultimately these biomarkers should enable staging of HIV disease, targeted development of candidate curative interventions, precise prediction of loss of remission, and the design of a low-cost detection device for use by the individual.

The underlying premise of the HIV Reservoirs Consortium is that successful development of an effective HIV cure will require a dramatically improved, multidimensional understanding of the rebound-competent viral reservoir and the ability of specific interventions to target this reservoir. This effort, in turn, is only likely to be accomplished by comprehensive, iterative, “whole body” *in vivo* analysis of ART-suppressed SIV/SHIV infection in nonhuman primate models, known to authentically recapitulate key relevant aspects of human HIV infection.

Since the vast majority of HIV-infected cells, harboring either actively replicating or latent virus, is tissue based,^32^ and since the pathobiology of the disease reflects the extensive heterogeneity of the immune system found within lymphoid and nonlymphoid tissues of the body, extensive sampling and analysis of these heterogeneous tissues throughout ART treatment and after ART discontinuation will be required to provide the necessary biologic characterization of the rebound-capable viral reservoir and its local regulatory/immune microenvironment(s). Such tissue sampling of well-defined infections during and after treatment is not feasible in humans. Moreover, interventions in humans must be safe and potentially therapeutic, whereas those in nonhuman primate models can be riskier and designed to either ameliorate or enhance disease, as long as justifiable to answer critical scientific questions.

Certain experiments can be conducted in humans. Thus, reservoir studies in humans will rely upon extensive postmortem analyses of tissue in areas of particularly high HIV prevalence (*e.g.*, KwaZulu-Natal in South Africa) as well as directed biopsies of gut and other lymphoid tissues of PLHIV on suppressive ART, using the same optimized assays developed for use in the nonhuman primate. Meanwhile, evaluation of effective immune responses against HIV will be carried out in carefully selected Exceptional Elite Controllers, that is, those with no plasma viremia after at least 10 years of documented infection in the absence of ART.^4,5^ Remarkably, some of the subjects already identified appear to have cured their infections based on the same criteria applied to the definition of cure in the “London patient”: undetectable plasma RNA levels, no replication competent virus, no detectable intact integrated provirus, and waning antibody responses.^4,5^

Properly executed, such studies in nonhuman primates and in humans are time consuming, painstaking, and highly resource intensive, requiring specialized infrastructure, expertise, and scientific approaches. Although critical for advancing the field, work of this nature does not offer predictable opportunities for the type of short- to medium-timeframe accomplishments that enable publications and the procurement or renewal of traditional grant funding in academia. Moreover, appropriately powered studies of this type in HIV-infected humans and SIV/SHIV-infected nonhuman primates require upfront resources that are typically beyond those available through most conventional funding mechanisms. For these reasons, we believe that the field needs a new and fresh approach, one committing sustained support that recognizes the inherent challenges and technical risks of an ambitious research program that, if successful, would be transformative. We hope that early progress in the HIV Reservoirs Consortium will catalyze such support far into the future.

## Expected Risks and Mitigations

There is no question that the above two areas of focus (*in vivo* targeting and editing to effect a “single-shot” cure for HIV disease and the HIV Reservoirs Consortium) carry considerable risks. Going into this work, a bottom-line reality must be accepted: the aspirational goal of safe, effective, and widely accessible *in vivo* gene therapy is daunting and may not be doable. To reach this goal, it will be important to excite and engage the best investigators in academia and the private sector, and to then patiently yet optimistically embrace the time-honored process of data-driven science.

The scientific and technical challenges of this goal are clear. It is likely that all of today's “best bets” for HIV cure will fail. Even if an *ex vivo* route to remission is discovered, existing and future technologies for durable delivery *in vivo* may be unable to translate that success into a single-shot cure. Remission may be lost with exposure to reinfection. If the duration of remission is relatively short and highly uncertain, it may be difficult to monitor for rebound in a cost-effective manner.

The proposed mitigation of these risks is a dedicated effort through the HIV Reservoirs Consortium to discover and develop circulating biomarkers of the rebound-competent reservoir that might be used to facilitate the generation and evaluation of novel HIV cure concepts, and ongoing engagement with the best technologies for durable delivery and detection. Although there is no way to predict how many cure concepts must be tested until one works, it is possible to establish a process more likely to assure success.

Another challenge is that an HIV cure intervention is likely to demand the integration of multiple cutting edge and likely valuable technologies that originate from different sources. This could create an intellectual property barrier and, at the least, presents a technical challenge. Investment in the HIV Frontiers Program is a step toward mitigating this intellectual property challenge by establishing a broad waterfront of rights to apply technologies for HIV cure in sub-Saharan Africa. One or more of those technology partners will be important to engage as an integrator of multiple technologies; notably, these companies are already aligned closely with and drawing intellectual property from leading academic laboratories in the field.

The development and implementation of curative interventions for sickle cell disease may provide an attractive model for developing important elements of HIV cure. However, its utility for HIV cure depends on the success of cure designs for sickle cell disease and the continued ability to leverage commercial interest. Technical failure or commercial exit would necessitate identification of alternative diseases where analogous cell targeting and editing techniques are implemented with applicability for sub-Saharan Africa. There are likely to be alternate opportunities in, for instance, oncology, although not as specific to sub-Saharan Africa as is the case of sickle cell disease.

An important assumption of the HIV Reservoirs Consortium, motivated by established observations, is that the nonhuman primate SIV/SHIV platform will be useful for understanding the HIV reservoir and human immune control, and that it will be effective in prioritizing interventions for clinical evaluation. Should this assumption be proven untenable under more intense scrutiny, the HIV Reservoirs Consortium could be adapted by scaling down components of the nonhuman primate platform and scaling up the human platform.

Finally, since the HIV Reservoirs Consortium differs from many collaborations previously established in the basic biological sciences, there is significant risk that it fails to maintain mission alignment, investigator commitment, and/or efficient resource allocation. This risk has been recently highlighted by restrictions on research imposed by the pandemic of SARS CoV-2. One mitigation tactic has been to engage in frequent and sustained dialog with the lead investigators of the Consortium. Another has been to maintain focus on the pursuit of a clear, although distant, goal: the discovery of circulating nonviral biomarkers that might then be further validated, optimized, and implemented.

## The Need for Partnerships

Given the extraordinary risks associated with the above goals, the long period of time that is likely to transpire before they are realized (or, conversely, shown to be beyond reach), and the resources required to both do the science and to incentivize those who do it, we have been eager to form partnerships in which complementary areas of expertise can be merged, resources combined, and risk shared (Fig. 2). Already, the HIV Frontiers Program has formalized a collaboration with the NIH focused on *in vivo* gene therapies for HIV and sickle cell disease.^61^ This collaboration fosters open communication, facilitates transparent review of strategically aligned programs, and unleashes additional resources for basic research on effective and safe targeting and editing approaches *in vivo*.

At the same time, efforts are being made by the HIV Frontiers Program to engage the focused expertise of small and medium-sized biotechnology companies that have cutting-edge technologies that can be applied to *in vivo* gene therapy. We are also working to cement partnerships with multinational pharmaceutical companies that have the expertise, resources, and motivation to bring such therapies into the field and through to commercialization. Should it be possible to align such partnerships on the common goal of affordable and accessible *in vivo* single-shot cures for HIV and sickle cell disease, it may then be the case that such therapies can be brought to all parts of the world.

Should such interventions prove to be clinically beneficial, it would also seem inevitable that similar methods will be aimed at multiple other diseases, infectious and otherwise. This would fundamentally shift the way in which we think about medical care and the infrastructures required for its provision. It might also make such care affordable and available for all, even in resource-replete parts of the world.
